# DOCTOR, I’M PREGNANT. Psychopharmacological treatment of depression in pregnant women. A clinical case of a pregnant woman and major depressive disorder

**DOI:** 10.1192/j.eurpsy.2023.2147

**Published:** 2023-07-19

**Authors:** M. Queipo De Llano De La Viuda, G. Guerra Valera, C. Vallecillo Adame, C. De Andrés Lobo, T. Jiménez Aparicio, M. Fernández Lozano, I. D. L. M. Santos Carrasco, N. De Uribe Viloria

**Affiliations:** Hospital clinico Universitario de Valladolid, Valladolid, Spain

## Abstract

**Introduction:**

Depression during pregnancy can appear with a prevalence of up to 11% of pregnant women. Psychotherapeutic treatment in these cases is considered the first option, but treatment with antidepressants is sometimes required in these cases.

**Objectives:**

To present a clinical case of a pregnant patient diagnosed with depression.

**Methods:**

Literature review of the psychopharmacological treatment of depression during pregnancy and possible complications.

**Results:**

A 25y Year old woman, 22 weeks pregnant, who lives with her partner. She has no background in mental health. Paternal aunt diagnosed with type I Bipolar Disorder. She goes to the Mental Health Center for evaluation, due to anxiety and depressive symptoms of 4 weeks of evolution, she refers sadness and apathy, continuous crying, somatic anxiety and obsessive ruminations in relation to childbirth and inability to care for your child. Suicidal ideation as a resolution of her discomfort. She presents with global insomnia and a significant loss of appetite, with a weight loss of 3 kg. Treatment with sertraline 50 mg/day was started, with good tolerance and clinical response

**Conclusions:**

The psychopharmacological treatment of antenatal depression is a challenge for the psychiatric professional. In all cases, an adequate balance must be made between the risks and complications for the fetus and the psychopathological stability of the pregnant woman. Among the main risks of untreated depression are: preterm delivery and low birth weight, an increased risk of suicide and alterations in the development during the baby’s infancy. The most used antidepressants are the SSRIs, with sertraline being a good option. Paroxetine has been associated with cardiac defects in the newborn. There are studies with tricyclics and duals but no specific teratogenic pattern has been seen. They are associated with an increased risk of spontaneous abortion. Exposure during the third trimester may be associated with obstetric complications.

**Disclosure of Interest:**

None Declared

